# Magnetic‐Responsive Carbon Nanotubes Composite Scaffolds for Chondrogenic Tissue Engineering

**DOI:** 10.1002/adhm.202301787

**Published:** 2023-09-17

**Authors:** Muthusamy Saranya, Aldeliane M. da Silva, Hanna Karjalainen, Geir Klinkenberg, Ruth Schmid, Birgitte McDonagh, Peter P. Molesworth, Margrét S. Sigfúsdóttir, Ane Marit Wågbø, Susana. G. Santos, Cristiana Couto, Ville‐Pauli Karjalainen, Shuvashis Das Gupta, Topias Järvinen, Luisa de Roy, Andreas. M. Seitz, Mikko Finnilä, Simo Saarakkala, Anne Marie Haaparanta, Lauriane Janssen, Gabriela S. Lorite

**Affiliations:** ^1^ Microelectronic Research Unit University of Oulu Oulu 90570 Finland; ^2^ Research Unit of Health Science and Technology University of Oulu Oulu 90220 Finland; ^3^ Department of Biotechnology and Nanomedicine SINTEF Industry Trondheim 7030 Norway; ^4^ Instituto Nacional de Engenharia Biomédica Instituto de Investigação e Inovação em Saúde Universidade do Porto Porto 4200‐135 Portugal; ^5^ Institute of Orthopedic Research and Biomechanics Center for Trauma Research Ulm University Medical Center Ulm 89081 Ulm Germany; ^6^ Askel Healthcare Ltd. Helsinki 00530 Finland

**Keywords:** carbon nanotubes, cartilage, magnetic stimulation, smart nanomaterials, stimuli‐responsive scaffold, tissue engineering

## Abstract

The demand for engineered scaffolds capable of delivering multiple cues to cells continues to grow as the interplay between cell fate with microenvironmental and external cues is revealed. Emphasis has been given to develop stimuli‐responsive scaffolds. These scaffolds are designed to sense an external stimulus triggering a specific response (e.g., change in the microenvironment, release therapeutics, etc.) and then initiate/modulate a desired biofunction. Here, magnetic‐responsive carboxylated multi‐walled carbon nanotubes (cMWCNTs) are integrated into 3D collagen/polylactic acid (PLA) scaffold via a reproducible filtration‐based method. The integrity and biomechanical performance of the collagen/PLA scaffolds are preserved after cMWCNT integration. In vitro safety assessment of cMWCNT/collagen/PLA scaffolds shows neither cytotoxicity effects nor macrophage pro‐inflammatory response, supporting further in vitro studies. The cMWCNT/collagen/PLA scaffolds enhance chondrocytes metabolic activity while maintaining high cell viability and extracellular matrix (i.e., type II collagen and aggrecan) production. Comprehensive in vitro study applying static and pulsed magnetic field on seeded scaffolds shows no specific cell response in dependence with the applied field. This result is independent of the presence or absence of cMWCNT into the collagen/PLA scaffolds. Taken together, these findings provide additional evidence of the benefits to exploit the CNTs outstanding properties in the design of stimuli‐responsive scaffolds.

## Graphical abstract

Magnetic‐responsive carbon nanotube/collagen/polylactic acid scaffolds are successfully fabricated. In vitro tests using cartilage cells (i.e., chondrocytes) demonstrate that the produced scaffold enhances chondrocytes metabolic activity while maintaining high cell viability and cartilage‐relevant extracellular matrix. The exposure to magnetic fields in the range of 200–800 mT does not affect the cell response. Adverse effect on cells is not observed.

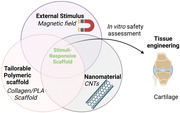

## 1 Introduction

Tissue engineering scaffolds have conventionally been developed to promote cell adhesion, maintain cell viability, facilitate nutrient, and growth factors transport and provide structural support for subsequent tissue development, while also ensuring biocompatibility and nontoxicity. Despite providing adequate micro‐ and macroenvironment for tissue regeneration, most of the conventional scaffolds lack or fail to provide natural cell signaling pathways, which limits their capabilities to regulate cell response. In recent years, stimuli‐responsive scaffolds have emerged as a strategy to modulate cell response and promote functional tissue regeneration, while also maintaining the required attributes of conventional passive scaffolds. Among other approaches to create stimuli‐responsive scaffolds, smart nanomaterials (NMs) commonly employed in the fields of electronics, energy, textile, and materials have been translated to tissue engineering applications.^[^ ^1^, ^2^, ^3^, ^4^ ^]^

Smart or stimuli‐responsive NMs have been integrated into polymeric matrices to act as nanotransducers to mediate and/or convert different types of energy into physical and chemical cues, inducing specific cell behavior such as cell orientation^[^ ^5^, ^6^ ^]^ and extracellular matrix (ECM) production.^[^ ^7^, ^8^ ^]^ Carbon nanomaterials are among the state‐of‐the‐art materials recently employed in drug delivery^[^ ^9^, ^10^ ^]^ and tissue engineering^[^ ^5^, ^11^, ^12^, ^13^, ^14^, ^15^, ^16^, ^17^, ^18^, ^19^ ^]^ research, offering the advantages of large surface area, mechanical strength, freedom of design, and good biocompatibility.^[^ ^19^, ^20^ ^]^ These materials also leverage the innate ability to respond to remote stimulation like electrical, photoacoustic, and magnetic fields. For example, graphene oxide (GO)‐based composites under stimulation^[^ ^18^ ^]^ by pH, light, heat, and magnetic field have been used for wound healing,^[^ ^17^, ^21^, ^22^ ^]^ cancer therapy,^[^ ^23^, ^24^ ^]^ and drug delivery^[^ ^25^, ^26^ ^]^ applications, while carbon dots have been studied as a luminescent nanomaterial for bone tissue engineering.^[^ ^16^ ^]^ Carbon nanotubes (CNTs) have also attracted the biomaterial scientist attention due to their outstanding mechanical, electrical, thermal, optical, and structural properties. CNTs either as templates (e.g., films, 3D micropillars) or as part of composites (e.g., hydrogels, scaffolds) have demonstrated their capability to provide topological, chemical, physical, and mechanical cues for long‐term survival of cells for functional tissue regeneration in case of fibroblast,^[^ ^27^ ^]^ neural,^[^ ^13^, ^14^, ^15^, ^28^, ^29^ ^]^ cardiac,^[^ ^5^, ^12^ ^]^ muscle,^[^ ^30^, ^31^ ^]^ and skeleton^[^ ^11^, ^19^ ^]^ tissue. On the other hand, the controversy on the use of CNTs in nanomedicine is well‐known in the scientific community due to the increasing concerns related to their possible long‐term toxicity.^[^ ^32^ ^]^ The fear of CNT in bioscience was elicited in 2008 when as‐produced CNTs induced asbestos‐like pulmonary pathogenicity after administrated in the abdominal cavity in mice.^[^ ^33^ ^]^ In 2019, CNTs were the first nanomaterials to be added to the so‐called SIN (“Substitute It Now”) list by the Swedish nonprofit organization ChemSec.^[^ ^34^ ^]^ Part of the scientific community reacted strongly as evidenced by several correspondences arguing against the “grouping all CNTs into a single substance category” and “banning CNTs would be a scientifically unjustified and damaging innovation.”^[^ ^35^ ^]^ Recently, a systematic review identified and analyzed 200 original publications on in vitro toxicological studies, showing that CNT dispersions at certain dose/incubation time are suitable for further studies in the field of targeted drug delivery, chemotherapy, tissue engineering, and implantable biosensors.^[^ ^36^ ^]^ A large variety of CNT types for biomedical applications exists offering a range of properties and biological behavior,^[^ ^37^ ^]^ which makes it hard to generally classify CNT as “toxic” or “nontoxic.” The possible CNT toxicity is highly dependent of their diameter, length, synthesis, and purification method as well as functionality.^[^ ^36^ ^]^ Thus, it is crucial to perform extensive evaluation of CNT toxicity for each individual proposed CNT‐based biomedical solution.

The challenges to restore articular cartilage from injuries or diseases are highly attributed to its limited self‐regenerative capacity associated with its avascular characteristic. The use of CNTs as part of composite scaffolds for cartilage repair has shown promising results, including enhancement of chondrocyte attachment and promotion of chondrogenic ECM expression.^[^ ^38^ ^]^ In another approach, magnetic field stimulation of articular chondrocytes has been studied as a possible therapeutic method of cartilage repair.^[^ ^39^, ^40^, ^41^, ^42^ ^]^ Pulsed electromagnetic field (PEMF) has been the most explored method to investigate the possible benefit of magnetic stimulation over articular chondrocytes in vitro.^[^ ^39^, ^40^, ^41^, ^42^ ^]^ The emphasis of the reported studies is on chondrocytes proliferation, metabolic activities, and, particularly type II collagen, aggrecan, and Sox9 expression^[^ ^39^, ^40^, ^41^, ^42^, ^43^, ^44^, ^45^ ^]^ via real‐time polymerase chain reaction. Overall, the reported findings of PEMF stimulation over chondrogenesis are to a great extend inconsistent. While some studies report modest improvement in cartilage‐related ECM expression,^[^ ^39^, ^40^, ^41^ ^]^ other studies show no significant effects.^[^ ^42^ ^]^ The reported discrepancies are mostly due to the difference in stimulation protocols, including type of PEMF device, PEMF parameters (exposure duration, amplitude, frequency, etc.), cell type, and cell‐seeding templates. In terms of PEMF parameters, PEMF studies have typically employed exposure duration from 10 min to 8 h per day and more consistently an amplitude around 2 mT. Interestingly, while one study reported an enhancement of cartilage‐relevant ECM by exposing the 3D pellet culture to 2 mT for 10 min day^−1^,^[^ ^39^ ^]^ another study showed that an 8 h day^−1^ is required to observe a significant enhancement on same gene expression.^[^ ^41^ ^]^ The later study also reported that a more effective way to achieve ECM expression enhancement was to use an innovative single‐pulsed electromagnetic field with 1T amplitude for 3 min day^−1^.^[^ ^41^ ^]^ The main difference between the two mentioned studies is the cell type, indicating that distinct cell types may respond differently to magnetic actuation. We here hypothesize that introducing magnetic‐responsive CNTs into an evidently efficient 3D scaffold for the use of cartilage repair and, combining it with remote magnetic field stimulation (static and pulsed), would boost the positive effects observed in the individual studies related either to CNTs or magnetic stimulation.

In this work, we report a magnetic‐responsive collagen/polylactic acid (PLA) scaffold containing multi‐wall CNTs (MWCNTs) aiming to modulate cell chondrogenic capacity. Magnetic‐responsive carboxylated MWCNTs (cMWCNTs) were previously synthesized, characterized, and homogenously integrated in hydrogel. Furthermore, the magnetic‐responsive cMWCNTs were aligned inside of the hydrogel via a remote magnetic field.^[^ ^46^ ^]^ Herein, the magnetic‐responsive cMWCNTs were introduced into collagen/PLA scaffolds via a filtration method. The pristine collagen/PLA scaffolds were provided by Askel Healthcare Ltd. (https://www.askelhealthcare.com/). While PLA is a Food and Drug Administration (FDA)‐approved safe polymer material (https://www.accessdata.fda.gov/cdrh_docs/pdf8/K082276.pdf), collagen is considered the most important biomaterials in connective tissue regeneration. The filtration method was optimized to ensure sample preparation reproducibility. The impact of cMWCNT introduction onto collagen/PLA scaffolds in terms of structure, thickness, porosity, and biomechanical performance is presented. Unlike most of the reported CNT‐based studies for tissue engineering, a comprehensive in vitro safety assessment has been performed beyond basic cell viability, ensuring that the proposed scaffold addresses the CNT toxicity concerns and can be used for further preclinical studies. After being cultured on cMWCNT/collagen/PLA scaffolds, the human chondrocytes metabolic activities were found to be significantly higher when compared to pristine collagen/PLA scaffolds, while maintaining cell viability and cartilage‐relevant ECM expression. In vitro evaluation of the scaffolds subjected to remote stimulation suggests that the cell response is independent of the applied magnetic field within the investigated parameters.

## 2 Results and Discussions

### 2.1 Fabrication and Characterization

The cMWCNT/collagen/PLA scaffolds are nanocomposite matrices in which cMWCNT were integrated into collagen/PLA scaffolds. Nonsterile collagen/PLA scaffolds were provided by Askel Healthcare Ltd. The collagen/PLA scaffolds are a composite structure of porous collagen and PLA mesh. These scaffolds were designed by Askel Healthcare Ltd. for the use of cartilage repair in weight‐bearing joints. The MWCNTs were produced via chemical vapor deposition (CVD) with a precursor of xylene and ferrocene mixture and further carboxylated as previously reported.^[^ ^46^ ^]^ The carboxylation was performed to enable homogenous dispersion of MWCNTs in water, which is a bio‐friendly solvent to be used with natural polymeric‐based scaffolds including collagen/PLA scaffolds. The obtained cMWCNTs were characterized by energy dispersive X‐ray spectroscopy (EDS), transmission electron microscope (TEM), and Raman microspectroscopy as detailed in the Supporting Information Figure S1. In addition, X‐ray photoelectron microscopy (XPS) was performed for pristine MWCNTs and cMWCNTs to determine the presence and concentration of the carboxylic groups on the CNTs surface (Figure S2, Supporting Information). The small amount metal oxide and metal carbonate peaks observed in pristine MWCNT is from the silicon oxide wafer surface and disappeared or reduced, respectively, after the acid treatment (Table S1, Supporting Information). The presence of the carboxylic groups is evidenced by the significant increase of atomic % of C═O bond in O 1s and the surge of O─C═O bond in C 1s. The observed change of atomic % for C─C bond in C 1s and C─O bond in O 1s when comparing pristine MWCNT and cMWCNT are due to the acid treatment, which removes amorphous carbon and creates defects on the MWCNT. Previously, we have shown that the Fe content of the synthesized cMWCNTs corresponds to 0.8% of the total mass of cMWCNTs by inductively coupled plasma mass spectrometry.^[^ ^46^ ^]^ The ferromagnetic behavior of both pristine MWCNTs and carboxylated cMWCNTs was also confirmed in previous work.^[^ ^46^ ^]^

**Figure 1:**
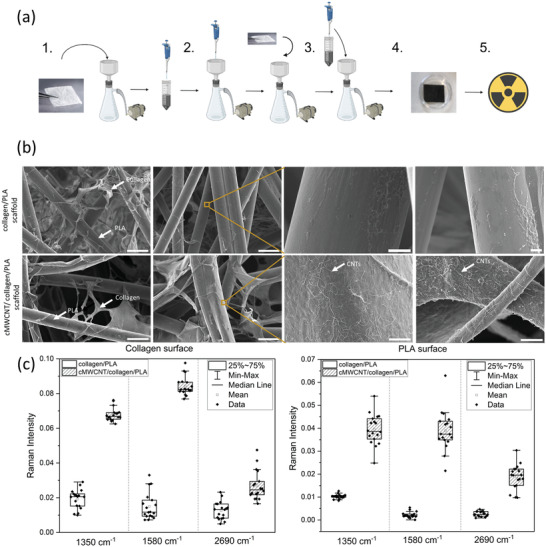
Fabrication and characterization of cMWCNT/collagen/PLA scaffolds. a) Schematic of integration of cMWCNT water‐based ink into the collagen/PLA scaffolds. Created with BioRender.com. b) SEM images showing CNTs, collagen, and PLA content in the scaffolds. c) Univariate analysis of Raman spectra evidencing the widespread presence of cMWCNTs on cMWCNT/collagen/PLA scaffolds.

The method to obtain highly homogenous cMWCNT water‐based ink was established for printing electronics^[^ ^47^ ^]^ and recently translated to the fabrication of aligned cMWCNT hydrogels.^[^ ^46^ ^]^ The reproducible protocol provides a final concentration of 0.2 mg mL^−1^ of homogenous cMWCNT water‐based ink. Concentrations higher than 0.2 mg mL^−1^ showed large precipitation and agglomerates formation. Different concentrations in the range of 0.02 to 0.1 mg mL^−1^ and several approaches were attempted to integrate the cMWCNT homogenously into collagen/PLA scaffolds without damaging their structure and affecting their key mechanical characteristics. Manually dipping (in/out) and immersing (10 to 30 min) the scaffold into the cMWCNT water‐based ink and immersion of the scaffold for 30 min into the cMWCNT water‐based ink followed by freeze drying did not end up to preferable results. The integration of cMWCNT into the collagen/PLA scaffolds was successfully achieved by filtrating the cMWCNT water‐based ink through the scaffold using a simple vacuum filtration setup. The scaffold integrity was visually maintained which was further confirmed during the scaffold physicochemical characterization. The steps to integrate cMWCNT into collagen/PLA scaffolds were optimized to ensure reproducibility at laboratory scale production. In summary, nonsterile collagen/PLA scaffolds as provided by Askel Healthcare Ltd. were placed on a porous filter connected to a vacuum pump (**Figure** **1**a, Step 1). A volume of 4 mL of cMWCNT water‐based ink at a concentration of 0.1 mg mL^−1^ was manually pipetted onto the scaffold surface (Supporting Information Video S1). The pipetting should be performed as coating the scaffold surface drop‐by drop in good speed and in both directions. Then, the scaffold was let to rest for 1 min and turned to the other side where the step 2 was repeated (Figure 1a, Step 3). The cMWCNT/collagen/PLA scaffold was dried at room temperature (RT) and later sterilized by gamma irradiation prior to in vitro studies (Figure 1a, Steps 4 and 5).

Scanning electron microscope (SEM) images of cMWCNT/collagen/PLA scaffolds showed that cMWCNTs appeared to coat both the fibrous PLA and collagen surfaces homogenously (Figure 1b). The presence of cMWCNTs was further verified by acquiring Raman spectra in 18 distinct spots on collagen and PLA surfaces of cMWCNT/collagen/PLA and control scaffolds (Supporting Information Figure S3). Univariate analysis of the Raman spectra corroborates with SEM images showing the presence of G (1580 cm^−1^) and D (1350 cm^−1^) bands in every measured spot on cMWCNT/collagen/PLA scaffolds in contrast to the absence of these bands on collagen/PLA scaffolds (Figure 1c). The reproducibility from sample to sample of the developed method was assessed by preweighting the scaffold before and after the integration of cMWCNTs using an analytical scale and assuming that the mass gain is a result from the incorporation of cMWCNTs. The average analytical values of the mass gain measured for 60 cMWCNT/collagen/PLA scaffolds were found to be in the range of 0.1–0.3 mg, with a 10% of the samples showing values out of this range (either lower or higher). The observed variation was considered acceptable not only due to the intrinsic error from the integration method (mostly handheld) but as well because the variability of the collagen/PLA ratio from sample to sample can play a role on how much cMWCNTs absorb on the scaffold.

Micro‐computed tomography (microCT) imaging was used to evaluate the collagen content, thickness, and porosity of the scaffolds. The collagen content was reduced by 42% after the cMWCNT integration into collagen/PLA scaffolds, indicating that part of the collagen is washed out during the fabrication process. The scaffold thickness was significantly reduced (34%) by the integration of cMWCNTs, which can be associated with the drying step in room temperature after passing the cMWCNT water‐based ink through the scaffold and as well the washed‐out collagen. cMWCNT/collagen/PLA scaffolds showed slightly reduced (2%) porosity in comparison to collagen/PLA scaffolds.

### 2.2 Biomechanical Performance

**Figure 2:**
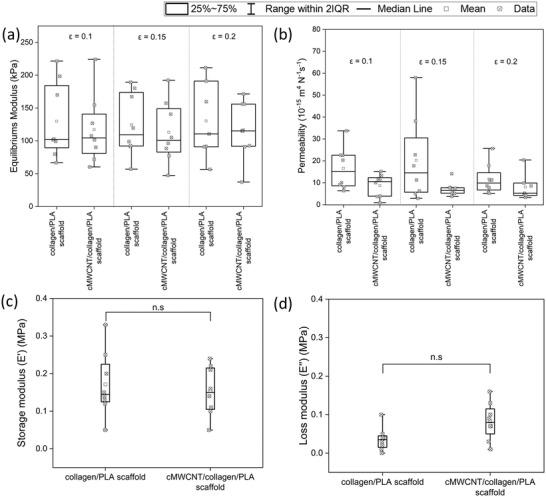
Biomechanical properties of cMWCNT/collagen/PLA scaffolds and collagen/PLA scaffolds. a) Equilibrium modulus (*E*_eq_) and b) hydraulic permeability of the tested scaffolds given in median values with range over the three tested strain rates (*ε* = 0.1; *ε* = 0.15; *ε* = 0.2). c) Loss modulus (*E*′) and d) storage modulus (*E*″) for both scaffolds, whereby each data point shows the mean value of the *n* = 10 applied cycles. Nonparametric statistical analyses: *n* = 8; *p < 0.05.

A comprehensive evaluation of the biomechanical performance of the cMWCNT/collagen/PLA scaffolds was performed by using a multistep confined compression relaxation testing, which is one of the most frequently performed methods to investigate the mechanical properties of articular cartilage samples and tissue engineered constructs.^[^ ^48^ ^]^ In articular cartilage, fluid pressurization and stress dissipation are time dependent because of its biphasic nature.^[^ ^48^, ^49^ ^]^ Constantly loading a cartilage sample and thereby compressing the tissue leads to an effusion of the interstitial fluid. Keeping the applied strain constant, the recorded load (stress) decreases (relaxation phase) until an equilibrium state is reached. At this end, no fluid flow within the tissue occurs anymore. Only the solid ECM (i.e., collagen fibers and proteoglycans) is responsible for the material properties.^[^ ^49^ ^]^ Therefore, the equilibrium modulus of articular cartilage represents the stiffness of the solid ECM. When testing the scaffolds, it can be assumed that the collagen, the PLA fibers, and integrated cMWCNTs define the material properties after reaching an equilibrium state. Based on this assumption, the matrix stiffness (*E*_eq_), hydraulic permeability (*k*), storage modulus (*E*′), and loss modulus (*“E”*) were investigated. At all studied strain rates (*ε* = 0.1, *ε* = 0.15, and *ε* = 0.2), no significant differences in *E*_eq_ were found between cMWCNT/collagen/PLA and collagen/PLA scaffolds (*p* > 0.05) (**Figure** **2**a). *E*_eq_ results also indicate that neither scaffold is dependent on the applied strain rate.

The resistance against fluid flow through the fiber network—, i.e., the hydraulic permeability *k*—in combination with the degree of tissue hydration is important to control the deformational behavior of articular cartilage. Because 3D scaffolds should be able to mimic the extracellular matrix, the permeability is also an important material property of scaffolds used for cartilage repair. Fluid flow through a solid matrix with a very low permeability would cause high frictional force for water flow, thus requiring large compressive loads to maintain the flow. In this case, the fluid pressure can provide a significant contribution in load support and therefore minimizing the stress acting on the solid phase.^[^ ^49^ ^]^ In articular cartilage, this fluid flow is often referred to as strain‐dependent permeability, since it becomes more difficult to squeeze fluid from cartilage with prolonged compression.^[^ ^49^ ^]^ During confined compression testing of the scaffolds, the phosphate‐buffered saline (PBS) within the 3D matrices was extruded out of the fiber network by applying a pressure on the samples by the porous indenter. When analyzing the water flow characteristics of both scaffolds, only minor influence of the strain rate on the hydraulic permeability was found (Figure 2b). The permeability of cMWCNT/collagen/PLA and collagen/PLA scaffolds were in the range of 5.29 and 15.23 10^−15^ m^4^ N^−1^ s^−1^ (Figure 2b). The collagen/PLA scaffolds revealed the highest values for *k* at each strain rate, within a range of 9.93–15.23 10^−15^ m^4^ N^−1^ s^−1^ (Figure 2b). However, the comparison of cMWCNT/collagen/PLA and collagen/PLA scaffolds indicated no significant difference (*p* > 0.05) between the scaffolds at none of the applied strain rates (Figure 2b).

Under dynamic conditions, cMWCNT/collagen/PLA and collagen/PLA scaffolds revealed similar (*p* > 0.05) storage moduli (*E*′) (Figure 2c). The loss modulus of the cMWCNT/collagen/PLA scaffold was 4% higher compared to the *E*″ of the collagen/PLA scaffolds, indicating no statistical difference (*p* > 0.99) (Figure 2d). For both scaffolds, the storage modulus was higher than the loss modulus, suggesting that the composite scaffold can be regarded as mainly elastic. Similar results were found in several studies investigating the viscoelastic properties of bovine and human articular cartilage by a dynamic mechanical analysis under gait‐relevant frequencies.^[^ ^50^ ^]^

Altogether these results let suggest that the integration of cMWCNT into collagen/PLA scaffolds did not compromise the biomechanical properties of the designed scaffold despite the observed changes in porosity, thickness, and collagen content by microCT.

### 2.3 In Vitro Safety Assessment

Endotoxin levels, bacterial contamination, and cytotoxicity were assessed to evaluate the potential risks of cMWCNTs for in vitro studies. The in vitro assessment was performed on cMWCNT water‐based ink and as well on cMWCNT/collagen/PLA scaffolds. The sterility was found <10 cfu mL^−1^ for both the samples, while endotoxin contents were <5 EU mL^−1^ and 0.006 EU unit^−1^ (1 unit in 4 mL water) for cMWCNT water‐based ink and cMWCNT/collagen/PLA scaffolds (*n* = 6), respectively. These values are lower than the acceptable regulatory limits for contamination showing no restrictions on the use of the scaffolds for further in vitro studies.

**Figure 3:**
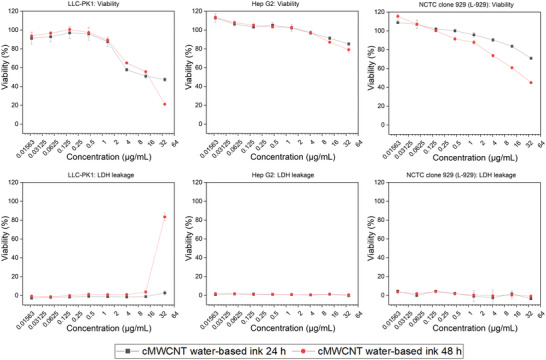
In vitro safety assessment of cMWCNT water‐based ink. MTT viability and LDH leakage in cell lines LLC‐PK1, Hep G2, and L‐929. The concentration of cMWCNT water‐based ink ranges from 0.02 to 37 µg mL^−1^ and exposed for 24 and 48 h.

The cMWCNT water‐based ink was assessed for potential induction of cytotoxicity in vitro using cell lines Hep G2 (Hepatocyte carcinoma), LLC‐PK1 (Porcine kidney), and NCTC clone 929 (L‐929, murine fibroblast). For the cytotoxicity analysis, the cMWCNT water‐based ink was diluted directly in cell‐specific medium to expose concentrations ranging from 0.02 to 37 µg mL^−1^. The results showed a dose‐dependently reduced cell viability for concentrations above 1.5 µg mL^−1^ for LLC‐PK1 and L‐929cell lines (**Figure** **3**). At 37 µg mL^−1^, the LLC‐PK1 viability was significantly lowered at the 48 h time point compared to the 24 h time point. Reduction of viability for L‐929 is more pronounced for cMWCNT concentrations above 4 µg mL^−1^ and after 48 h exposure. Hep G2 viability is reduced for cMWCNT concentrations higher than 12 µg mL^−1^ and no significant difference was observed when comparing 24 and 48 h exposure times. These results indicate that Hep G2 is significantly less sensitive to cMWCNT water‐based ink than LLC‐PK1 and L‐929. Increased lactate dehydrogenase (LDH) values were observed only for LLC‐PK1 at the highest cMWCNT concentration (37 µg mL^−1^) and 48 h of exposure, indicating significant cell death and loss of membrane integrity at this concentration (Figure 3).

For the cytotoxicity analysis of cMWCNT/collagen/PLA scaffolds, viability and LDH leakage data were calculated as percentages (%) of the negative (only cell medium, viability) and positive controls (Triton X100, *n* = 3, LDH leakage), and a reduction of viability or increase of LDH of more than 20% is considered as a significant response. In this case, extracts from the cMWCNT/collagen/PLA scaffolds were diluted in respective cell culture medium and exposed to the study cell lines. In contrast to the results for cMWCNT water‐based ink, no significant cytotoxicity was detected for all tested cMWCNT/collagen/PLA scaffolds.

### 2.4 Macrophage Pro‐Inflammatory Polarization

**Figure 4:**
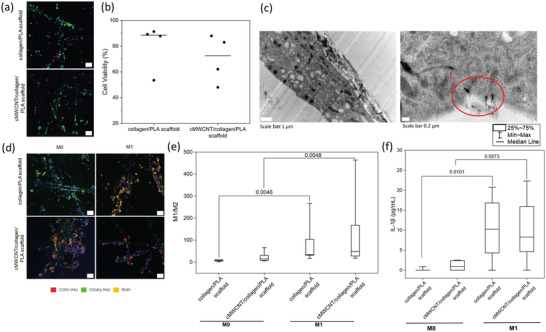
Macrophage polarization on cMWCNT/collagen/PLA and collagen/PLA scaffolds. a) Live (green)/dead (red) staining for the scaffolds showing few dead cells. Scale bar 50 µm. b) Quantification of cell viability with LDH release. c) SEM images showing macrophage adhesion to cMWCNT/collagen/PLA scaffolds and cMWCNT internalization by cells (red circle and arrows). d) Confocal images showing expression of CCR7 and CD163 on unstimulated (M0) and stimulated scaffolds (M1). Scale bar 50 µm. e) Quantification of M1/M2 markers for M0 and M1 condition. f) Quantification of IL‐1β level in supernatants of M0 and M1 condition of scaffolds.

The potential risk of the cMWCNTs to induce a pro‐inflammatory response was investigated by evaluating the impact of culture on cMWCNT/collage/PLA scaffolds for macrophage polarization, in comparison with culturing on collage/PLA scaffolds. Macrophages are central cells in the host response to biomaterials and modulating their polarization from M1 to M2 is considered to promote inflammation resolution and progression to tissue repair/regeneration.^[^ ^51^ ^]^ Our previous results, using a different polymeric scaffold, indicate that primary human macrophages polarization can be modulated by biomaterial composition and has similar response to in vivo macrophages.^[^ ^52^ ^]^ Human primary monocyte‐derived macrophages were differentiated directly on the scaffolds, resulting in a very long exposure time to cMWCNTs. Calcein acetomethoxy (AM) staining was performed to visualize the live cells. As shown in **Figure** **4**a, the majority of macrophages remain viable (green) after 13 days, with few dead macrophages (red), which is expected for long‐term cultures. LDH release in culture supernatants was also quantified to assess cell viability (Figure 4b). Despite a decrease in cell viability for some macrophage donors, there were no significant differences between macrophages cultured on cMWCNT/collagen/PLA scaffolds compared to collagen/PLA scaffolds. MWCNTs are reported to be internalized by macrophages and able to, in a concentration‐dependent fashion, act as adjuvants in a pro‐inflammatory stimulation, leading to increased cytokine secretion.^[^ ^45^ ^]^ TEM micrographs indicate a good adhesion of macrophages to cMWCNT/collagen/PLA scaffolds and cMWCNT internalization by the cells (Figure 4c). However, no pro‐inflammatory adjuvant effect upon M1 stimulation.

Next, we investigated macrophage activation by analyzing the expression of CCR7, as a M1 marker (i.e., pro‐inflammatory‐activated macrophages) and CD163, as a M2 marker (i.e., anti‐inflammatory or alternatively activated macrophages) using confocal microscopy imaging (Figure 4d). As expected, a predominance of green staining (CD163) was observed on collagen/PLA scaffolds in unstimulated conditions (Figure 4d, M0), with an increase of red staining (CCR7) when cells were activated with lipopolysaccharide (LPS) and IFN‐γ (Figure 4d, M1). Macrophages cultured on cMWCNT/collagen/PLA scaffolds indicated more frequently a double CCR7 and CD163 staining in unstimulated conditions (M0) when compared to unmodified scaffolds or with stimulated conditions (M1) (Figure 4d). The images were further analyzed by counting the macrophages positive for each marker across different donors and calculating the M1/M2 ratio. The calculated M1/M2 ratio indicated significant differences for M0 versus M1 conditions for each scaffold type, as expected (Figure 4e). However, no significant differences for M1/M2 ratio were observed when comparing cMWCNT/collagen/PLA with collagen/PLA scaffolds (Figure 4e). To further investigate the macrophage pro‐inflammatory response, levels of IL‐1β were quantified in culture supernatants. IL‐1β levels significantly increased from unstimulated M0 to stimulated M1 conditions as expected, while no significant increases were observed on cMWCNT/collagen/PLA versus control scaffolds (i.e., collagen/PLA scaffolds).

Taken together, these results indicated that the presence of cMWCNT does not increase macrophage activation per se and does not act as an adjuvant when macrophages are exposed to pro‐inflammatory M1 conditions.

### 2.5 In Vitro Cell Response

To understand the use of cMWCNT/collagen/PLA scaffold as a vehicle to provide local stimuli, chondrocytes were seeded on scaffolds and subjected to an external magnetic field. In our preliminary study, we investigated the effect of a static magnetic field at 400 and 800 mT on chondrocytes seeded on cMWCNT/collagen/PLA and collagen/PLA scaffolds (Supporting Information Figure S4). These magnitudes were selected based on previous study, in which the alignment of the synthesized cMWCNT is induced by magnetic field exposure.^[^ ^46^ ^]^ Based on literature survey,^[^ ^39^, ^40^, ^41^ ^]^ the time of exposure duration was set as 5 min day^−1^. In this first preliminary study, the cells were cultured for 7 days, with time points of 3 and 7 days. Cell metabolic activity was evaluated via the Prestoblue assay. The expression of type II collagen and aggrecan by the cells was evaluated via immunohistochemistry as these are major components of ECM of articular cartilage and are regulated by chondrocytes. Chondrocytes showed significantly increased metabolic activity for cMWCNT/collagen/PLA scaffolds when comparing with collagen/PLA scaffolds, indicating higher cell attachment and proliferation due to the presence of cMWCNTs. No significant differences on cell metabolic activity were observed in dependency with the applied field of 400 and 800 mT. For all the conditions, a slight decrease in chondrocyte metabolic activity was observed from 3 D to 7 D. The cell viability was maintained, and aggrecan/collagen II expression was observed for all studied conditions and time points.

**Figure 5:**
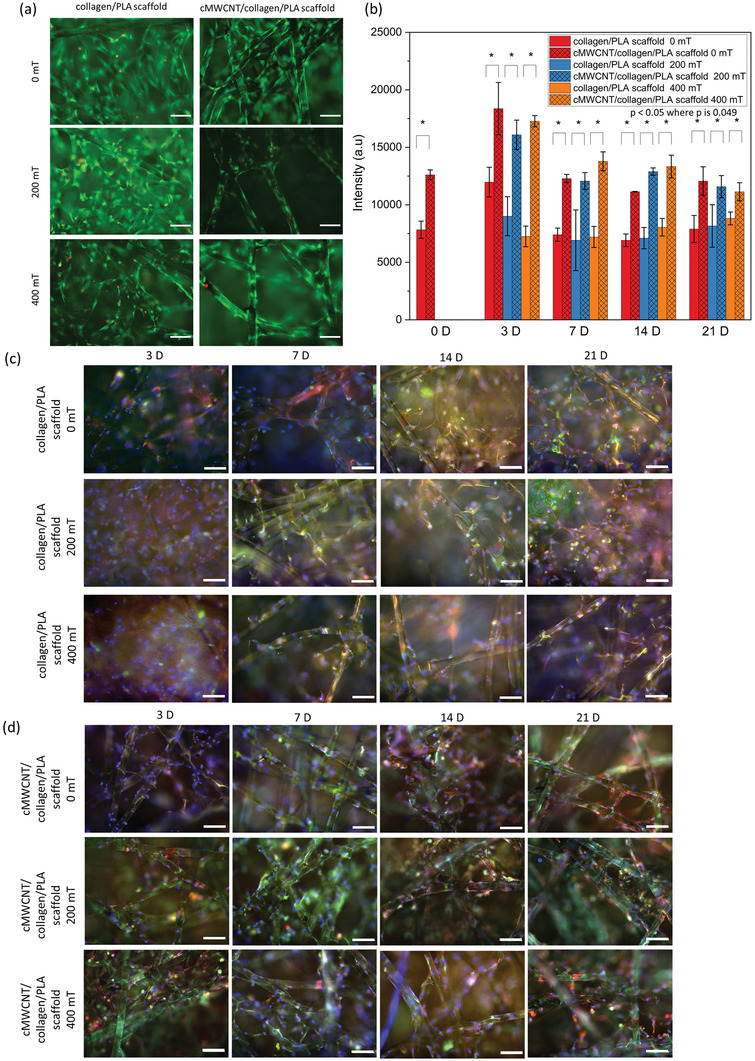
Cell response on scaffolds through static magnetic field. a) Representative live/dead images of collagen/PLA and cMWCNT/collagen/PLA scaffolds on 7 D at different magnetic field strengths (200 and 400 mT) and unstimulated condition (i.e., 0 mT). Scale bar 100 µm. b) Cell metabolic activity for the scaffolds, where * means *p* < 0.05. Statistical analyses were performed with Kruskal–Wallis ANOVA with post hoc Dunn's test. c,d) Representative immunostaining images. Chondrocytes were stained with 4′,6‐diamidino‐2‐phenylindole (DAPI, blue), collagen II (green), and aggrecan (red). Scale bar 100 µm.

**Figure 6:**
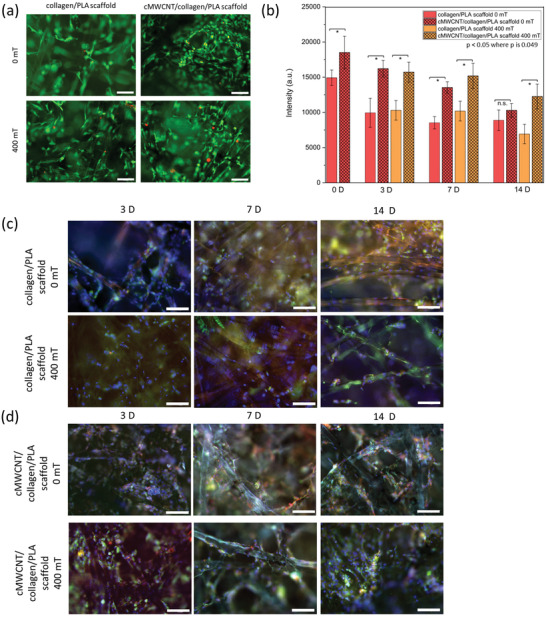
Cell response on scaffolds through pulsed magnetic field. a) Representative live/dead images of collagen/PLA and cMWCNT/collagen/PLA scaffolds on 7 D at 0 and 400 mT. Scale bar 100 µm. b) Cell metabolic activity for the scaffolds, where * means *p* < 0.05. Statistical analysis was performed with Kruskal–Wallis ANOVA with post hoc Dunn's test. c,d) Representative immunostaining images. Chondrocytes were stained with DAPI (blue), collagen II (green), and aggrecan (red). Scale bar 100 µm.

To further investigate whether the cMWCNT/collagen/PLA scaffold in combination with magnetic field stimuli affects cell behavior, we performed a comprehensive series of experiments including unstimulated condition (i.e., 0 mT) in experiments with a static magnetic field (**Figure** **5**) and experiments with a ramping magnetic field (**Figure** **6**). A magnetic field of 400 mT was included in all the experiments for comparison with the preliminary data (Supporting Information Figure S3). The pulsed magnetic field was generated by modulating the power supply of the magnet with signal generator, creating total of six pulses during the 5 min day^−1^ stimulation (Supporting Information Figure S5). All obtained results corroborate the preliminary data findings with great reproducibility. High cell viability was observed for all conditions, by live/dead staining (Figures 5a and 6a), indicating that no adverse effect is observed due to the presence of cMWCNTs or due to the exposure to different magnetic fields. The expression of type II collagen and aggrecan was visually detected for all studied conditions and time points (Figures 5c,d and 6c). Significantly higher cell metabolic activity was observed when chondrocytes were seeded on cMWCNT/collagen/PLA scaffolds compared to the collagen/PLA scaffolds for all time points and studied conditions (Figures 5b and 6b), except for the unstimulated (i.e., 0 mT) condition at 14 D used as control during the pulsed magnetic field experiment set (Figure 6b). In the latter case, a slight difference is still observed between the two scaffolds, but it is not statistically significant. The cell metabolic activity showed a different trend on unstimulated samples from 0 D to 3 D when comparing the two parallel experiments; while in one experiment an increase in metabolic activity is observed (Figure 5b), a decrease is showed in the other one (Figure 6b). This might be explained by the different cell line source used in each of the two experiments. While comparing 3 D to 7 D samples, the cell metabolic activity either significantly decreased or no significant changes were observed. In this case, no trend was observed in relation to the scaffold type, presence/absence of stimulation, and/or magnetic field type. Except by the unstimulated cMWCNT/collagen/PLA scaffolds used as control for the pulsed magnetic field, no significant changes on cell metabolic activity were observed from 7 D to later time points for the studied conditions. In case of the unstimulated cMWCNT/collagen/PLA scaffolds used as control for the pulsed magnetic field, a decrease in cell metabolic activity is observed from 3 D to 14 D. However, the cell metabolic activity levels were still higher (3 D and 7 D) or similar (14 D) in this condition when comparing the control scaffolds (i.e., collagen/PLA).

Collectively, these results indicated that the presence of cMWCNT in the scaffolds promote cell attachment and proliferation while boosting metabolic activity, whereas no relevant cell response in dependency with the applied magnetic field (either static or pulsed) for both scaffolds. In contrast to the reported PEMF studies,^[^ ^39^, ^40^, ^41^ ^]^ our experiments were carried out using static or pulsed magnetic fields with strength in the range of 200–800 mT. The lack of effect due to the physical stimuli on the chondrogenesis can be related to several factors at play such as: the dynamic of human chondrocytes culture, limited scope of cell analysis at molecular level, not sufficient concentration of cMWCNT and iron oxide NPs on the scaffold, and limited range of the explored stimulation parameters (i.e., time of exposure, magnitude/type of magnetic field). Furthermore, here magnetic‐responsive cMWCNTs were employed as a vehicle in the attempt to enhance local stimulation. Similarly, magnetic nanoparticles (MNPs) have been used to provide magneto‐mechanical stimulation of the cells.^[^ ^43^, ^44^ ^]^ The combination of MNPs triggered by magnetic field has shown a more significant effect in the expression of markers associated with chondrogenesis,^[^ ^53^ ^]^ degrees of anisotropy in scaffold structure to orient cell seeding,^[^ ^54^ ^]^ and as well advantages of remote controlling the stimuli with spatial and/or temporal precision.^[^ ^55^ ^]^ On the other hand, MNPs mostly need to be covered with a biocompatible polymer to be stabilized in physiologic fluids, avoid aggregation, and to provide surface for additional functionalities.^[^ ^56^, ^57^ ^]^ The absence of the these might result in a negative impact on the cell viability, metabolic activity, and proliferation,^[^ ^43^, ^58^, ^59^ ^]^ which is not the case for the magnetic‐responsive cMWCNTs presented here.

## 3 Conclusion

The translation of CNT‐based scaffolds as stimuli‐responsive medical devices to clinical use will foster comprehensive data on CNTs as a biomedical product including in vitro safety assessment, manufacturing freedom, and responsiveness to external stimuli. In this study, magnetic‐responsive cMWCNTs were synthesized and integrated into collagen/PLA scaffolds via an easy but highly reproducible filtration‐based method. The developed method allowed widespread adsorption of cMWCNTs in both components (i.e., collagen and PLA) of the pristine scaffold. After cMWCNT integration, the collagen content and scaffold thickness were reduced whereas the biomechanical performance remained the same. The structural changes caused by the cMWCNT integration (i.e., reduced collagen content, scaffold thickness, and porosity) did not affect the scaffold capabilities to support cell attachment and proliferation. Compared with the pristine collagen/PLA scaffolds, the proposed cMWCNT/collagen/PLA scaffolds enhanced chondrocytes metabolic activity while maintaining high cell viability and cartilage‐relevant ECM (i.e., type II collagen and aggrecan) expression. Neither cytotoxicity nor macrophage pro‐inflammatory response was detected during in vitro safety assessment of cMWCNT/collagen/PLA scaffolds, supporting the further application of cMWCNTs as integrated component of biomaterials. In contrast with our initial hypothesis, no effects on cell response were observed due to the combination of the magnetic‐responsive cMWCNTs/collagen/PLA scaffolds and external magnetic field. The use of magnetic‐responsive cMWCNT as a vehicle to provide local stimuli in combination with remote magnetic field stimulation is a new attempt and should not be excluded yet. Instead, a different set or combination of parameters for the stimulations could be explored and/or applied with other cell types. For instance, longer exposure time to the magnetic field was not in the scope of this work. In addition, extensive cell analysis at molecular level could reveal effects not observed via immunostaining imaging and metabolic activity assays. Furthermore, CNTs are well known for their outstanding conductivity and capability to absorb near‐infrared light and, therefore, the cMWCNT/collagen/PLA scaffolds also represent a promising platform to be explored with electrical and photoacoustic stimulation for several tissue engineering applications.

## 4 Experimental Section

### Synthesis of cMWCNT

The cMWCNTs were prepared as the previous publications.^[^ ^46^ ^]^ Briefly, MWCNTs were synthesized via CVD in a quartz tube reactor. The reactor inlet was consisted of a feeding tube with copper hair ball connected to a syringe, temperature probe, and argon gas line and the outlet was connected to vacuum and a water pump. A catalyst precursor ferrocene (Fe(C_2_H_5_)_2_) was dispersed in a carbon precursor xylene ((CH₃)₂C₆H₄)) at a concentration of 20 g L^−1^. The precursors were introduced at 96 mL h^−1^ until the copper hair looked wet, then the rate was changed to 6 mL h^−1^ for 60 min and the precursors were transported by argon (Ar) at atmospheric pressure to facilitate the synthesis of MWCNT on the Si/SiO_2_ chips placed in the reactor at 770 °C. The obtained MWCNTs were grinded and functionalized with carboxylic groups in an acid blend of 3:1 of nitric acid and sulfuric acid. The mixture was sonicated for 5 h at room temperature (RT), followed by repeated centrifugation (4000 rpm for 15 min) to replace the acid with deionized (DI) water. The carboxylated MWCNTs (cMWCNTs) were filtered and washed on a 0.2 µm cellulose nitrate membrane until neural pH was reached, there after they were dried overnight at 80 °C.

### Preparation and Characterization of cMWCNT Water‐Based Ink

cMWCNTs dispersion (1 mg mL^−1^) was prepared in sterile water and sonicated for 30 min at room temperature. The supernatant was collected after each centrifugation at 4000 rpm for 15 min and repeated four to five times until no precipitate was visually observed. The concentration was determined by polyvinylidene fluoride filter paper with known volume of dispersed cMWCNTs. The cMWCNT water‐based ink was evaluated according to the previous publication.^[^ ^46^ ^]^ In short, the morphology and elemental composition were characterized by TEM and EDS, respectively, on JEOL JUM 2200FS TEM. A few drops of MWCNT water‐based ink were suspended in equal drops of ethanol and casted on copper mesh covered with Holey Carbon Film (HC300‐Cu, Electron Microscopy Science). The outer diameter of 100 individual cMWCNTs was measured using EM software Beta 0.85 (Teitz Video and Image Processing Systems GmbH). Data were represented as column graph using OriginPro2020b. The diameter mean of cMWCNT was done with Lorentz fitting and the result was represented as *d* ± *σ*, where *d* is the center and *σ* is the standard deviation calculated as width/2. For Raman microspectroscopy, a confocal Raman imaging microscope system (DXR2xi, Thermo Fisher Scientific, USA) equipped with a 10×/0.25 NA objective, a 50 µm confocal pinhole aperture, and a wide range grating (50–3250 cm^−1^, spectral resolution: 5 cm^−1^) was used to collect signal from cMWCNTs. Raman signal was excited by a 532 nm laser (5 mW) and the spectra were collected after 0.1 s of photobleaching, using an exposure time of 0.5 s and 20 accumulations. The pristine MWCNT and cMWCNT were characterized by XPS (Thermo Fisher Scientific Escalab 250 XI system with Al Kα X‐ray source) and the data evaluation was done by Avantage software.

### Characterization of the cMWNCT/Collagen/PLA and Pristine Collagen/PLA Scaffolds

The surface morphologies of the scaffolds were observed by field‐emission scanning electron microscopy (FESEM, Zeiss Ultra Plus). The charge up effect was minimized by sputtered coating (Agar High Resolution Sputter Coated 208 HR) with platinum (30 s, 40 mA).

To analyze the distribution of cMWCNT on the scaffolds, Raman spectral measurements were performed on cMWCNT/collagen/PLA scaffolds and collagen/PLA scaffolds. With optical light microscopy, the laser was focused on the different parts of the scaffold structure (i.e., collagen and PLA) to study the cMWCNT adsorption. Raw Raman spectra were truncated into the 350–3200 cm^−1^ region and subjected to noise removal by a 5‐point moving average filter. The spectral baseline was corrected by subtracting a third degree polynomial function from the raw spectra and finally the spectra were vector normalized. All spectral preprocessing steps were performed using in‐house scripts written in MATLAB (MathWorks Inc., MA, USA). The heights of the cMWCNT‐specific bands are calculated to demonstrate the presence of cMWCNTs in the scaffolds.

To evaluate the thickness, collagen content, and porosity, scaffolds (*n* = 6) were imaged with a micro‐computed tomography (µCT) desktop device (SkyScan 1272, Bruker microCT, Kontich, Belgium). For image acquisition, the source voltage was kept at 40 kV, the source current at 250 µA, and no additional filtering was used. Integration time was set to 700 ms with frame averaging of 4 to collect 1200 projections. Cross‐sectional images with isotropic voxel size of 1.0 µm were reconstructed using the NRecon software (Bruker microCT) with beam‐hardening and ring‐artifact corrections being applied. In addition, another set of images was produced with single distance phase‐retrieval reconstruction with a delta‐beta ratio of 300 to better separate PLA scaffold from collagen.^[^ ^60^ ^]^ Measurements were done with CTAn software (Bruker microCT). Before the analysis, general image processing methods were applied to remove noise from the image stack. Porosity was measured as total porosity (open and closed pores). The thickness of scaffolds was calculated from the middle of the scaffold.

### Biomechanical Performance of the Scaffolds

Extensive evaluation of the biomechanical performance of both scaffolds was performed using a material testing machine (Z10, ZwickRoell, Germany) (Supporting Information Figure S6). Two cylindrical samples with a diameter of 4.8 mm were punched out of the scaffolds using a commercial biopsy punch (Stiefel Laboratories Inc., UK). Both scaffolds were placed one above the other in a testing chamber filled with PBS providing confined conditions. A porous ceramic (Al_2_O_3_) cylinder was placed on top of the samples to ensure a free uniaxial fluid flow through the 3D matrices. The materials testing machine equipped with a stainless‐steel punch induced a previously defined strain amplitude to the samples, while the resulting force was measured by a 20 N load cell (ZwickRoell, Germany). An initial preload was applied to ensure the same testing conditions at the beginning of each test, while the thickness of both stacked samples (*h*_0_) was automatically registered. Because viscoelastic properties depend on the extent of the applied compression, the samples were tested under three consecutive strain levels of *ε*_i_ = 0.1, 0.15, 0.2 with a strain‐dependent loading rate of 3% *h*_0_ min^−1^ to load each sample equally.^[^ ^61^ ^]^ The applied strain levels represented physiological (10% and 15%) and pathological (20%) conditions.^[^ ^62^, ^63^ ^]^ Each strain level was held constant for 60 min (relaxation time) to ensure an equilibrium state was reached. Directly after the third strain level measurements were finished, a cyclic loading test was performed to simulate dynamic conditions. During ten cycles, a sinusoidal strain rate of *ε*_max_ = 0.25 at the physiological gait frequency of 1 Hz was applied on the samples and the force response was measured.

Data evaluation was performed using MATLAB R2020a (MathWorks Inc., USA). Two characteristic biomechanical parameters for viscoelastic materials were determined analyzing the multistep relaxation data. At each strain rate, the equilibrium's modulus *E*_eq_ as a measure of the matrix stiffness was calculated by the quotient of the stress at equilibrium (σ_(*t* → ∞)_) and the applied strain (*ε*_i_) (Equation (1))

(1) $$E_{\mathrm{eq}}=\sigma_{\left(t\to \infty \right)}/\varepsilon_{i};\;\varepsilon_{i}=0.1;0.15;0.2$$

The hydraulic permeability *k* as a measure of the resistance against fluid flow through the fiber network was calculated by solving the diffusion equation from Mow et al. by nonlinear least square regression at all three strain rates, respectively (Equation (2))^[^ ^49^ ^]^

(2) $$\sigma_{t}=\sigma_{t\to \infty }+2.H.\varepsilon_{i}.e^{\left({\left(-\pi /h_{0}\right)}^{2}.H.k.t\right)}$$

From the cyclic testing, the storage modulus *E*′ (Equation 3) and loss modulus *E*″ (Equation (4)) were determined. *E*′ relates to the stiffness or elastic behavior of a material and represents the energy stored in the structure. *E*″ represents the dissipation of energy due to internal friction and therefore relates to the viscous material behavior. δ is the determined phase difference between the stress and strain data.^[^ ^64^ ^]^ For each scaffold, the mean *E*′ and *E*″ values were calculated by averaging the determined *E*′ or *E*″ values of the ten applied cycles

(3) $$E^{\prime }=\frac{\sigma_{0}}{\varepsilon_{0}}\cos\delta$$

(4) $$E^{\prime \prime }=\frac{\sigma_{0}}{\varepsilon_{0}}\sin\delta \;=E^{\prime }.\tan\delta$$

### Endotoxin Content and Bacterial Contamination

The cMWCNT water‐based ink (0.37 mg mL^−1^) was diluted (e.g., 10‐, 100‐, 1000‐, 10 000‐, and 100 000‐fold) in endotoxin free water (Lonza kit) for evaluation of endotoxin content and in sterile PBS (Oxoid, BR0014) for bacterial contamination analysis, respectively. The lowest sample dilution gave recovery values between 50% and 100% for a known endotoxin spiking concentration of 0.5 EU mL^−1^ that were considered valid and applied to calculate sample endotoxin values. These dilutions were tested for endotoxin content with the PyroGene Recombinant Factor C kit from Lonza (Article number 50–658) according to the protocol described in the kit. All the equipment applied was classified as endotoxin free. For bacterial contamination, the test was done by plating on 3M Petrifilm Aerobic Count Plates (Article number 6400/6406/6442, 3M, St. Paul, MN). 1 mL from each dilution was subjected onto the Petrifilms. The samples were incubated at 35 °C for 72 h before the number of visible colonies on the Petrifilms was counted. Petrifilms with more than 200 colonies were not counted.

Endotoxin content and bacterial contamination for cMWCNT/collagen/PLA scaffolds were determined similarly as cMWCNT water‐based ink. The scaffolds were extracted overnight at RT in endotoxin free water according to FDA guidelines.^[^ ^65^ ^]^ The FDA guideline recommends that 10 units should be extracted in 40 mL water. However, due to the small size and the limited supply of the sample scaffolds, three parallel extractions of sample scaffolds in 4 mL of endotoxin free water were applied for each sample type. The scaffold extracts were tested for endotoxin content with the PyroGene Recombinant Factor C kit from Lonza (Article number 50–658). For testing the bacterial contamination, scaffold extracts were plated on 3M Petrifilm Aerobic Count Plates (Article number 6400/6406/6442, 3M, St. Paul, MN) and diluted in PBS as mentioned for MWCNT water‐based ink. 1 mL extract was subjected onto the Petrifilms and was incubated at 35 °C for 72 h before the number of visible colonies on the Petrifilms was counted. Petrifilms with more than 200 colonies were not counted.

### Cytotoxicity Assessment

cMWCNT water‐based ink of concentration ranging from 0.02 to 0.37 µg mL^−1^ was diluted directly in cell specific medium and tested after 24 and 48 h of exposure in the cell lines HepG2 (hepatocarcinoma), LLC‐PK1 (Porcine kidney), and NCTC clone 929 (murine fibroblast), with reduction of 3‐(4,5‐dimethyl‐2‐thiazolyl)‐2,5‐diphenyl‐2*H*‐tetrazolium bromide (MTT, viability) and release of lactate dehydrogenase (LDH) enzymes (membrane integrity) as readouts. The assays were performed according to the European Nanomedicine Characterisation Laboratory (EUNCL) SOPs EUNCL‐GTA01, EUNCL‐GTA02 and EUNCL‐GTA03 (https://euncl.org/). A Tecan robotic system was applied for the exposure procedure and a Beckman coulter robotic system with an integrated SpectraMax i3X plate reader from molecular devices was used to determine the release of cytosolic LDH caused by membrane rupture (LDH), and for quantification of metabolic activity by viable cells (MTT).

The cMWCNT/collagen/PLA scaffolds were extracted according to ISO‐10993‐5 in Medium 199, Roswell Park Memorial Institute (RPMI) 1640, and Dulbecco's modified Eagle medium cell culture medium for 24 h at 37 °C (one extraction in medium for each of the cell lines tested). Due to the small size of the scaffolds (5 mm) and the limited availability of scaffolds for testing, the extraction volume given in ISO‐10993‐5 could not be used. Therefore, a ratio of 750 µL medium per scaffold was applied in the extractions. Similar tests to MWCNT water‐based ink were performed, i.e., 24 and 48 h of exposure in the cell lines HepG2, LLC‐PK1, and L929 with the reduction of MTT (viability) and release of LDH enzymes (membrane integrity) as readouts.

### M1/M2 Macrophage Polarization

Human monocytes were isolated from buffy coats (BC) of health blood donors by negative selection, using a previously described method.^[^ ^66^ ^]^ In brief, BC was centrifuged for 20 min at RT and 1200 *g* (no brake), for blood component separation. Peripheral blood mononuclear cells (PBMCs) were collected and incubated with RosetteSep Human Monocyte Enrichment Cocktail (Stem‐Cell Technologies) for 20 min at RT under slow orbital agitation. The cell mixture was then diluted with an equal volume of 2% heat‐inactivated fetal bovine serum (FBS, Biowest) in PBS, carefully layered over Histopaque‐1077 (Sigma‐Aldrich Co.), and centrifuged for 20 min at RT and 1200 *g* (no brake). The enriched monocyte layer was collected and washed at least three times with PBS by centrifugation for 7 min at RT and 1300 rpm until the supernatant was clear. The cell pellet was resuspended in complete RPMI 1640 media with L‐glutamine (Corning) supplemented with 100 U mL^−1^ penicillin and 100 µg mL^−1^ streptomycin (1% P/S, both from Invitrogen) and 10% heat‐inactivated FBS (Biowest).

The cMWCNT/collagen/PLA and pristine collagen/PLA scaffolds were cut into 4 mm diameter disks using sterile biopsy punchers. Before cell seeding, scaffolds were incubated at 37 ^○^C in complete RPMI media for 1 h. Monocytes were seeded directly on top of the scaffolds at a density of 1 × 10^6^ cells per scaffold and cultured in complete RPMI media supplemented with 50 ng mL^−1^ of M‐CSF (ImmunoTools), at 37 °C in a humidified 5% CO_2_ incubator for 13 days. At 7 D, the cell culture media was replaced, and M‐CSF was removed. At 10 D, media was replaced, and when required cultures were stimulated with 10 ng mL^−1^ LPS, *Escherichia coli* O55:B5 (Sigma‐Aldrich Co.), and 50 ng mL^−1^ IFN‐γ (ImmunoTools) for M1 macrophage activation. Supernatants were collected under sterile conditions at 13 D, centrifuged at 10 000 rpm for 5 min at 4 °C (Eppendorf Centrifuge 5810R, VWR International LLC) to remove any cellular debris and stored at −80 °C until further analysis. Cells were used for the assays below.

To access macrophage viability, scaffolds were stained with 5 µL mL^−1^ Calcein‐AM in PBS for 45 min at 37 °C followed by washing and incubation with 5 µL mL^−1^ propidium iodide in PBS for 5 min at 37 °C. It was visualized using confocal laser scanning microscope (Leica SP5) using the Leica Application Suite X (LAS X) software. Images were processed with Fiji (ImageJ), ilastik for pixel classification, and CellProfiler for cell counting.

To check the adherence of macrophage to cMWCNT/collagen/PLA scaffolds and internalization of cMWCNTs by the cells, scaffolds were fixed using 2.5% glutaraldehyde/2% paraformaldehyde in cacodylate buffer 0.1 m (pH 7.4), post‐fixed with 1% osmic acid, dehydrated, and then embedded in epon resin (TAAB). Semi‐thin sections of the scaffolds with 50 nm thickness were prepared on an RM Ultramicrotome (PowerTome, Labtech) using a diamond knife. Sections were stained with uranyl acetate and lead citrate and electron micrographs were obtained using a Jeol‐1400 TEM (JEOL USA, Inc.) operating at 200 kV in 200 mesh size formvar carbon‐supported copper grids coupled with an Orius SC 1100 W digital camera (Orius). TEM was equipped with an EDS system to trace intracellular iron associated with cMWCNTs.

To test the number of macrophage positive for M1 and M2, on 13 D the scaffolds were washed and fixed with PFA 4% for 15 min. After fixing and washing, they were treated with Triton X‐100 0.2% (Sigma‐Aldrich) in PBS for 15 min at RT for cell membrane permeabilization. Then, samples were blocked with bovine serum albumin (BSA) 1% (VWR International LLC) in PBS for 1 h at RT to reduce nonspecific background staining. Finally, samples were incubated with primary antibody rabbit anti‐human CCR7 (M1 marker) (Abcam) at a 1:100 ratio over night at 4 °C. Then incubated with secondary antibody Alexa Fluor 647 goat anti‐rabbit (Thermo Fisher Scientific) at a 1:300 ratio for 1 h at RT, protected from light. Samples were then washed and blocked again with BSA 1% in PBS for 1 h at RT followed by incubation with primary antibody mouse anti‐human CD163 (M2 marker) (Bio‐rad) at a 1:100 ratio at 4 °C and later incubated with secondary antibody Alexa Fluor 594 goat anti‐mouse (Thermo Fisher Scientific) at a 1:300 ratio for 1 h at RT. The samples were visualized by confocal laser scanning microscope (Leica SP5) using Leica Application Suite X (LAS X) software. Images were processed with Fiji (ImageJ). The number of macrophages positive for M1 and M2 was counted across five images per scaffold and normalized for the total number of cells. Then the M1/M2 ration was calculated. A total of five independent experiments, with different macrophage donors were performed.

LDH release was assessed using the CytoTox96 assay (Promega, Madison, WI, USA), following the manufacturer's instructions. The presence of LDH was measured in cell free culture supernatants and absorbance (490 nm) was recorded in a microplate reader Synergy MX (BioTek, Winnoski, VT, USA). Total LDH release was achieved by using the LDH positive control provided by the CytoTox96 assay. Percent cell viability was calculated by subtracting the percentage cytotoxicity (percentage of released LDH relative to maximum LDH activity from the LDH positive control). The concentration of IL‐1 β produced by macrophages cultured on cMWCNT/collagen/PLA scaffolds and its control was quantified using the ELISA MAX Deluxe Set Human IL‐1 β kit, according to manufacturer's instructions (Biolegend), measured at 450 nm using a multiplate reader (Synergy Mx, BioTek).

### In Vitro Magnetic Stimulation Studies

Human chondrocytes from Cell application (402‐05A) between passage P4–P5 were used for the in vitro studies. All scaffolds were wetted with 100 µL cm^−2^ and incubated in an atmosphere of 5% CO_2_ and 95% air at 37 °C for 30 min prior to cell seeding. 650 000 cells in 50 µL cell suspension per 0.6 cm^2^ were seeded dropwise on the scaffold to ensure widespread seeding of the cells on the scaffolds. The cells were let to adhere to the scaffolds in the incubator for 2 h after which the scaffolds were incubated with 1 mL of human chondrocytes media (Cell applications). For the magnetic stimulation, the scaffolds were placed on custom‐made glass‐cuvette (diameter 2 cm) for the ease to move and stimulate the samples. Unstimulated scaffolds were placed in a 24 well plate.

The magnetic field stimulation setup was consisted of two electromagnetic coils and a power supply, providing a static magnetic field strength up to 1T. A magnetometer probe was used to monitor the magnetic field between the two electromagnetic coils. The power supply (Agilent 6674A, 0–60 V/0‐35A, DC power supply) parameters were adjusted to achieve 200, 400, and 800 mT according to each planned experiment. For the pulsed magnetic field, the output of the power supply was modulated with a signal generator (Agilent 33120A 15 MHz, Function/Arbitory waveform generator). The scaffolds were subjected to the magnetic field 5 min day^−1^. Unstimulated samples are marked as 0 mT, meaning that they were not exposed to any kind of magnetic field.

Cell metabolic activity was evaluated via the Prestoblue cell viability reagent (Thermo Fisher Scientific, Invitrogen, USA) at 0, 3, 7, 14, and 21 days. Note that 0 D means 24 h after cell seeding and prior to the daily magnetic field stimulation being applied. Before adding the Prestoblue solution, the scaffolds were lifted to a new well and washed once with PBS. A dilution of 1:10 v/v from the Prestoblue reagent and Chondrocyte Growth Medium was added to the scaffolds (500 µL). Samples were incubated for 1 h at 37 °C and 2 × 100 µL of the medium from each well was collected into a black‐wallet 96 well plate. As a blank sample, Prestoblue reagent in Chondrocyte Growth Medium without cells was used. A Wallac Victor3 multilabel reader (PerkinElmer, Waltham, MA, USA) was used to measure fluorescence intensity at a wavelength for excitation 544 nm and emission 615 nm. The metabolic activity of the cells was analyzed using three independent samples replicated per time point.

Chondrocytes viability on both scaffolds was determined by the LIVE/DEAD viability/cytotoxicity kit for mammalian cells (L3224, Molecular Probes Inc., Invitrogen) as per the instructions. The live cells were stained with Calcein AM whereas the dead cells were stained with Ethidium homodimer‐1. The images were taken by a Leica DM4B with a CoolLED pE300Q Lightsource and processed using Fiji ImageJ software.

To visualize the production of chondrocyte specific markers type II collagen and aggrecan, the scaffold were fixed for 15 min in 4% PFA, incubated in blocking buffer (10% NDS + 1% BSA + 0.1% Triton X‐100 in PBS) for 1 h at RT. Scaffolds were stained overnight at 4 °C with primary antibodies: collagen II (1:200, NB600‐844, Novus Biologicals) and aggrecan (5 µg mL^−1^, AF1220, R&D systems) diluted in blocking buffer, followed by incubation with secondary antibodies: donkey anti‐goat (1:200, NL557, R&D systems, orange/red, Ex/Em 557/574 nm) and rabbit anti‐mouse (1:200, NB7543, Novus Biologicals, green, Ex/Em 495/519 nm) for 1 h. The nuclei were stained with DAPI (1:2000, NBP2‐31156, Novus Biologicals) for 10 min and stored in PBS until observation.

### Statistics Analysis

For microCT and in vitro magnetic stimulation studies, a normality test was performed resulting in a nonnormal distribution using OriginPro2021 (OriginLab Corp.). Differences between the cMWCNT/collagen/PLA scaffolds and collagen/PLA scaffolds were investigated by Kruskal–Wallis with post hoc Dunn's test. The statistical significance level was set to *p* < 0.05.

For biomechanical evaluation, normal distribution was checked via the Shapiro–Wilk test, resulting in nonnormally distributed data. Analysis was performed using GraphPad Prism 7.03 software (GraphPad Software Inc., San Diego, United States). Differences between the cMWCNT/collagen/PLA and collagen/PLA scaffolds were investigated by Mann–Whitney testing. The statistical significance level was set to *p* < 0.05.

Confocal images for macrophage polarization were analyzed using ImageJ (Fiji). Statistical analysis on the images was performed using GraphPad Prism 8.3.2 (GraphPad Software, Inc.). Normal distribution was checked using Shapiro–Wilk normality test. As normal distribution of data was not verified, statistical comparisons were performed using Wilcoxon paired test for comparing two groups and nonparametric Friedman test, followed by Dunn's, for multiple comparisons, to compare three or more groups. A *p* < 0.05 value was considered statistically significant.

### Ethics Statement

Human primary monocytes were isolated from surplus buffy coats from healthy blood donors, kindly donated by Serviço de Imunohemoterapia, Centro Hospitalar Universitário de São João (CHUSJ), Porto. Sample collection and experimental protocols were performed in agreement with the principles of the Declaration of Helsinki and following the approval and recommendations of the CHUSJ Ethics Committee for Health (reference 90/19). Written informed consent was obtained from all subjects before sample collection. Samples were anonymized and no patient identification was provided to researchers.

## Conflict of Interest

The authors declare no conflict of interest.

## Supporting information

Supporting Information

supplemental video 1

## Data Availability

The data that support the findings of this study are available from the corresponding author upon reasonable request.
