# Supplementary material for: Magnetic‐Responsive Carbon Nanotubes Composite Scaffolds for Chondrogenic Tissue Engineering
Source: Adv Healthc Mater. 2023 Sep 17;12(30):2301787. doi: 10.1002/adhm.202301787 (PMC11468560; doi:10.1002/adhm.202301787)
Supplement: Supplementary file 1 — Supporting Information [file ADHM-12-2301787-s002.pdf]

ADVANCED HEALTHCARE MATERIALS

Supporting Information

for *Adv. Healthcare Mater.*, DOI 10.1002/adhm.202301787

Magnetic-Responsive Carbon Nanotubes Composite Scaffolds for Chondrogenic Tissue Engineering

*Muthusamy Saranya, Aldeliane M. da Silva, Hanna Karjalainen, Geir Klinkenberg, Ruth Schmid, Birgitte McDonagh, Peter P. Molesworth, Margrét S. Sigfúsdóttir, Ane Marit Wågbø, Susana. G. Santos, Cristiana Couto, Ville-Pauli Karjalainen, Shuvashis Das Gupta, Topias Järvinen, Luisa de Roy, Andreas. M. Seitz, Mikko Finnilä, Simo Saarakkala, Anne Marie Haaparanta, Lauriane Janssen and Gabriela S. Lorite**

Supporting Information

Magnetic Responsive Carbon Nanotubes Composite Scaffolds for Chondrogenic Tissue Engineering

Muthusamy Saranya[1], *Aldeliane M. da Silva*[1], *Hanna Karjalainen*[2], *Geir Klinkenberg*[3], *Ruth Schmid*[3], *Birgitte McDonagh*[3], *Peter P. Molesworth*[3], *Margrét S. Sigfúsdóttir*[3], *Ane Marit Wågbø*[3],*Susana. G. Santos*[4,5], *Cristiana Couto*[4,5], *Ville P. Karjalainen*[2], *Shuvashis Das Gupta*[2], *Topias Järvinen*[1], *Luisa d. Roy*[6], *Andreas. Seit*[6], *Mikko Finnilä*[2], *Simo Saarakkala*[2], *Anna Marie Haaparanta*[7] *and Gabriela S. Lorite*[1*]

[1]Microelectronics Research Unit, University of Oulu, Finland,[2] Research Unit of Health Sciences and Technology, University of Oulu, Finland [3]Department of Biotechnology and Nanomedicine, SINTEF Industry, Norway, [4] Instituto Nacional de Engenharia Biomédica, Universidade do Porto, Portugal,[5]Instituto de Investigação e Inovação em Saúde, Universidade do Porto, Portugal, [6]Institute of Orthopedic Research and Biomechanics, Ulm University, Germany, [7]Askel Healthcare Ltd, Helsinki, Finland

Corresponding Author

*Email: gabriela.lorite@oulu.fi

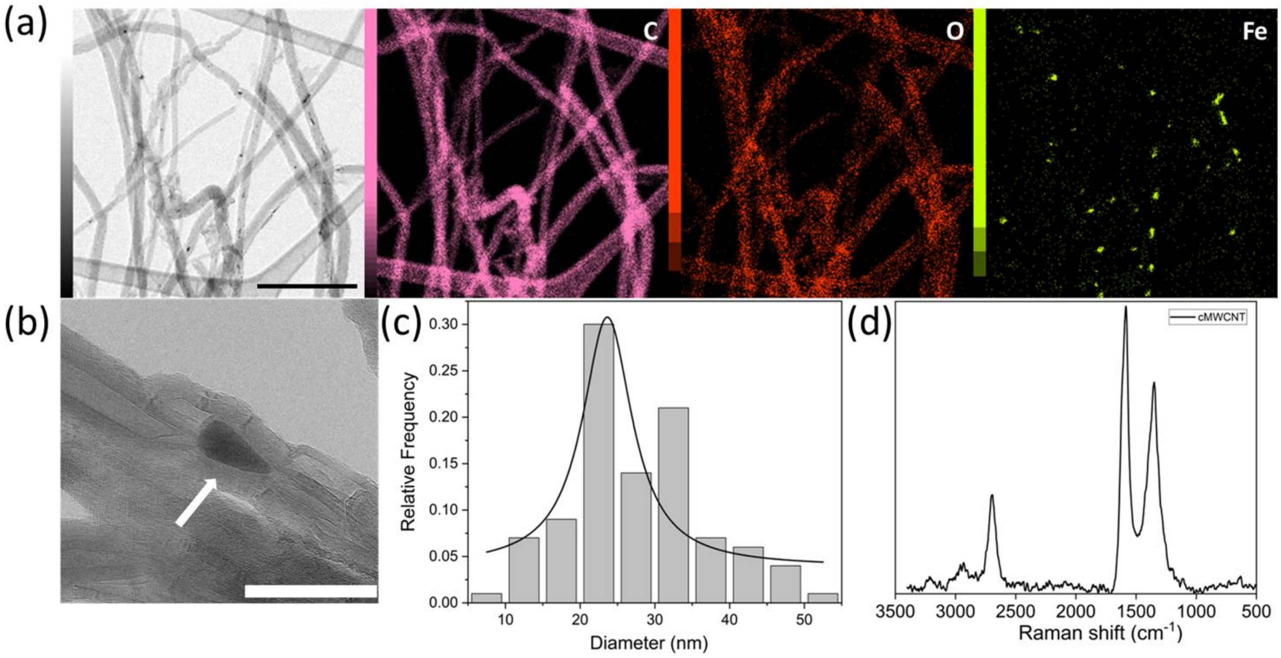

Figure S1: Characterization of cMWCNTs. (a) EDS of cMWCNTs confirming the presence of carboxylated groups (i.e. carbon and oxygen elements) and Fe deposition within the cMWCNT walls. Scale bar 0.5 µm. (b) TEM images of cMWCNTs, arrow showing Fe nanoparticles inside the hollow tube of cMWCNTs. Scale bar 40 nm. (c) Diameter distribution from a series of TEM images of cMWCNTs (23 ± 8 nm). (d) Raman spectrum of cMWCNT with D, G and G' bands of MWCNTs at 1380 cm^{-1}, 1580 cm^{-1} and 2690 cm^{-1}, respectively.

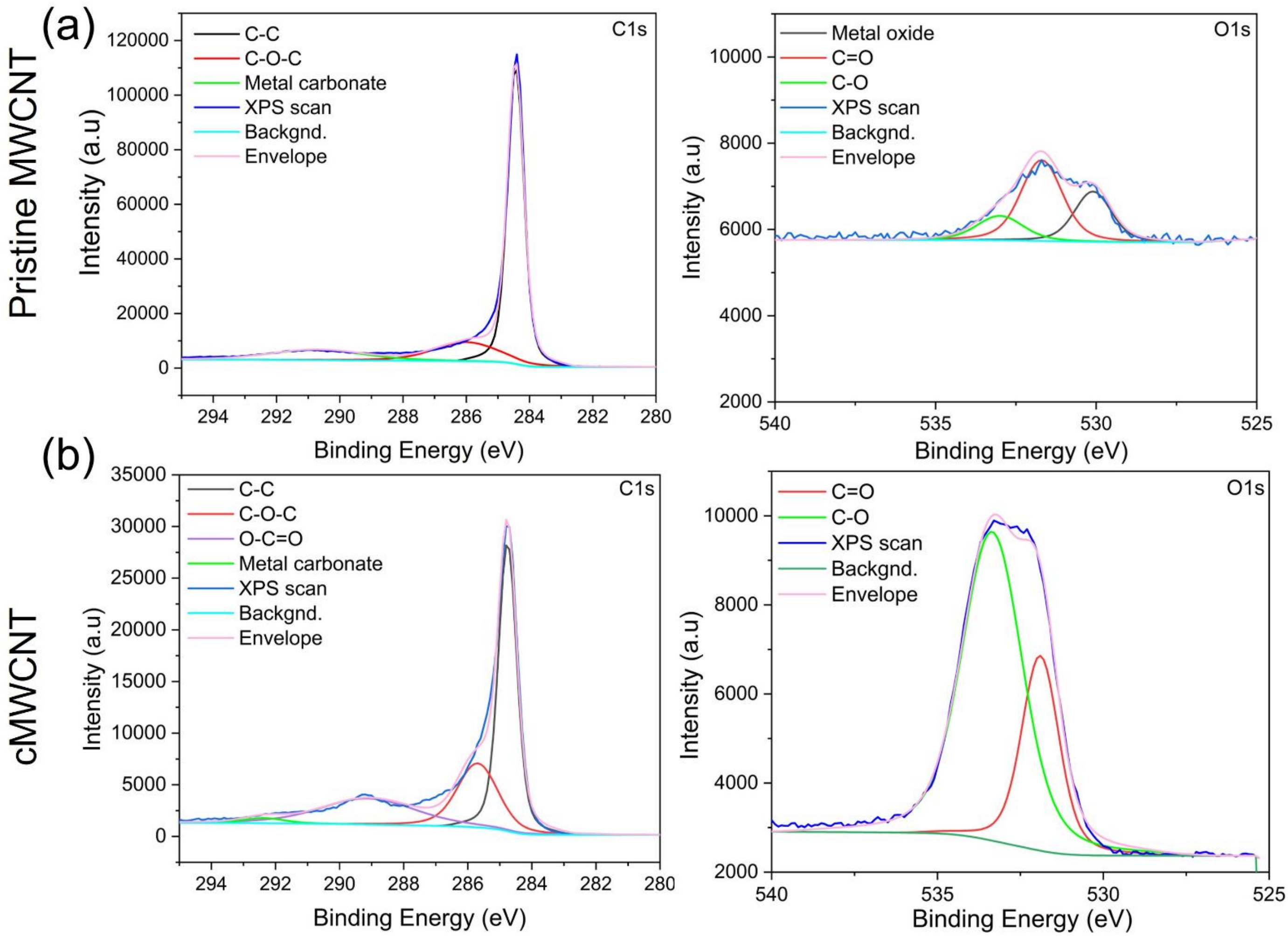

Figure S2: XPS analysis of pristine MWCNT and cMWCNTs. (a) Fitted XPS spectra C 1s and O 1s peak for pristine MWCNT. (b) Fitted XPS spectra of C 1s and O 1s of peak for cMWCNT.

Table S1: XPS analysis results of pristine MWCNT and cMWCNT

Element	Sample	Chemical state	Peak position (eV)	Atomic concentration (%)
C1s	Pristine MWCNT	C-C	284.44	65.23
	cMWCNT		284.76	31.67
	Pristine MWCNT	C-O-C	286	16.41
	cMWCNT		285.69	15.44
	Pristine MWCNT	O-C=O	-	-
	cMWCNT		289.12	14.71
	Pristine MWCNT	Metal carbonate	290.73	12.72
	cMWCNT		292.38	1.27
O1s	Pristine MWCNT	Metal oxide	530.1	1.64
	cMWCNT		-	-
	Pristine MWCNT	C=O	531.7	2.95
	cMWCNT		531.9	10.38
	Pristine MWCNT	C-O	533	1.05
	cMWCNT		533.35	26.53

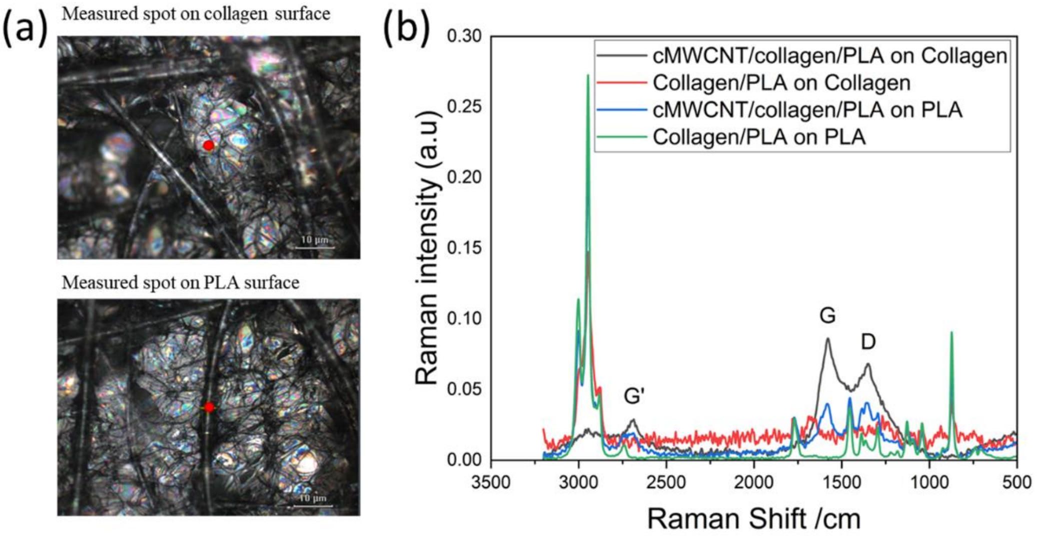

Figure S3: (a) Optical light microscope illustrated the measured spot-on collagen and PLA surfaces of the scaffolds. (b) Representative Raman spectra acquired on collagen and PLA surfaces from collagen/PLA and cMWCNT/collagen/PLA scaffolds showing the presence of the D (1380cm^{-1}), G (1580 cm^{-1}) and G' (2690 cm^{-1}) bands of CNTs only on the cMWCNT/collagen/PLA scaffolds.

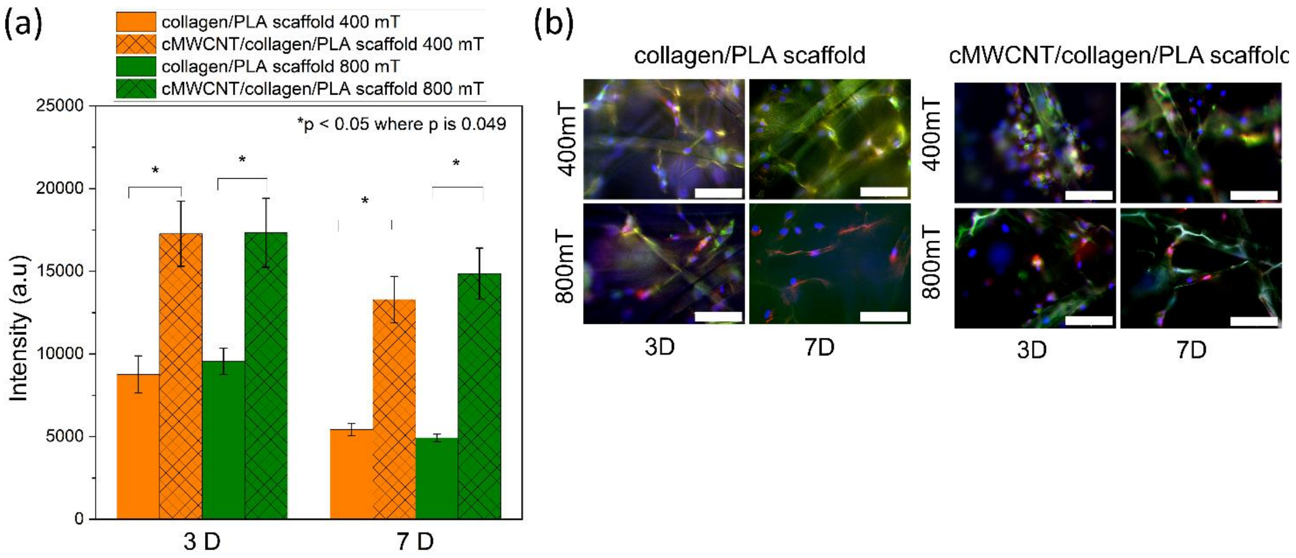

Figure S4: *In vitro* cell response on stimulated cMWCNT/collagen/PLA scaffolds through a static magnetic field. (a) Cell metabolic activity of the stimulated collagen/PLA and cMWCNT/collagen/PLA scaffolds at 3D and 7D, where * means $p < 0.05$. Statistical analysis performed with Kruskal-Wallis ANOVA with post hoc Dunn's Test. (b) Representative immunostaining images of chondrocytes cultured on collagen/PLA scaffolds and cMWCNT/collagen/PLA scaffolds stimulated 5 min/day with a static magnetic field (400 mT and 800 mT) for 3 and 7 days.

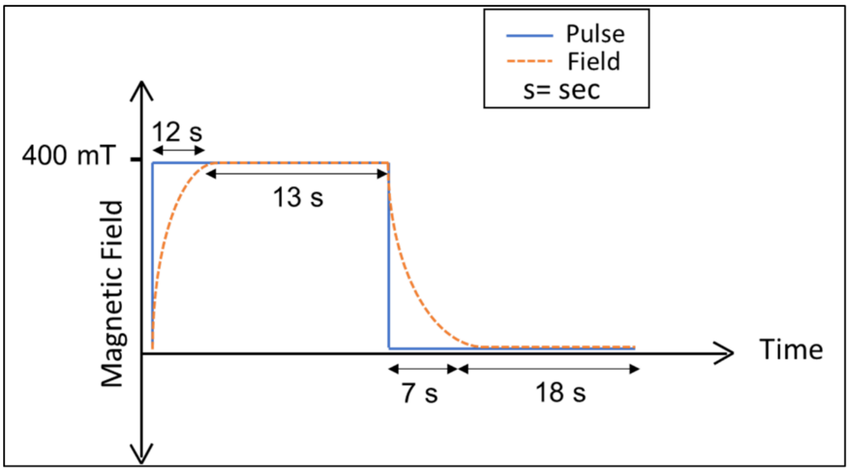

Figure S5: Modulating pulse from the signal generator and respective profile of the pulsed magnetic field.

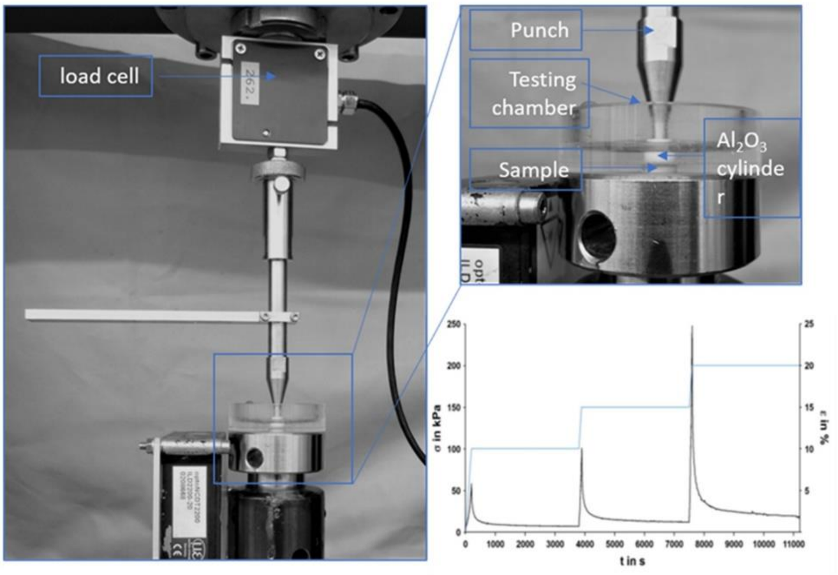

Figure S6: Biomechanical testing setup.

Video 1. Step by step of the cMWCNT integration method into collagen/PLA scaffold.
